# Post-it notes to improve questionnaire response rates in RCTs - findings from a randomised sub-study

**DOI:** 10.1186/1745-6215-16-S2-P102

**Published:** 2015-11-16

**Authors:** Ada Keding, Helen Lewis, Kate Bosanquet, Simon Gilbody, Hannah Buckley, Helen Tilbrook, David Torgerson

**Affiliations:** 1 University of York, York, UK

## Background

Valid treatment effect estimates in the analysis of RCTs using patient reported outcomes depend on adequate response rates. Evidence from non-RCT surveys suggests that inexpensive post-it notes may improve response rates.

## Aim

To assess the effectiveness of a post-it note attached to questionnaires on patient response rate and time to response in a mental health study population.

## Methods

A two-arm RCT was embedded into the follow-up of older adults as part of the UK Casper Plus trial and Casper Cohort. At 4 months follow-up, participants were randomised to receive either a questionnaire or a questionnaire + post-it note requesting completion. Logistic regression and time-to-event analyses were used to assess attrition. Results were combined with those of a previous embedded RCT (ATLAS) in a meta-analysis.

## Results

266 of 297 (89.6%) participants returned their questionnaire in the post-it note arm, compared with 282 of 314 (89.8%) in the control arm (OR 0.97, 95% CI 0.58 to 1.64, p=.92). The pooled OR of response rates from the meta-analysis was 0.94 (95% CI 0.65 to 1.37, p=.76). An exploratory analysis showed that participants with major depression were more likely, and participants with sub-threshold depression less likely to respond to post-it notes (p of interaction =.02). There was no evidence for group differences for the time taken to respond (p=.54) or whether a reminder was required (p=.80).

## Conclusions

Post-it notes were not found to be effective in improving retention in this trial. However, beneficial effects for sub-groups of clinical populations may exist.

